# Dose finding and *O*^6^-alkylguanine-DNA alkyltransferase study of cisplatin combined with temozolomide in paediatric solid malignancies

**DOI:** 10.1038/sj.bjc.6602740

**Published:** 2005-08-23

**Authors:** B Geoerger, G Vassal, F Doz, J O'Quigley, M Wartelle, A J Watson, M-A Raquin, D Frappaz, P Chastagner, J-C Gentet, H Rubie, D Couanet, A Geoffray, L Djafari, G P Margison, F Pein

**Affiliations:** 1 Department of Pediatrics, Institut Gustave Roussy, Villejuif, France; 2 UPRES EA 3535 ‘Pharmacology and New Treatments in Cancer’, Institut Gustave Roussy, Villejuif, France; 3 Institut Curie, Paris, France; 4 Information Systems, Institut Gustave Roussy, Villejuif, France; 5 Cancer Research-UK Carcinogenesis Group, Paterson Institute for Cancer Research, Manchester, UK; 6 Centre Léon Bérard, Lyon, France; 7 Hôpital d'Enfants, Nancy, France; 8 Hôpital de La Timone, Marseille, France; 9 Hôpital Purpan, Toulouse, France; 10 Department of Radiology, Institut Gustave Roussy, Villejuif, France; 11 Hopital Lenval, Department of Radiology, Nice, France; 12 Schering-Plough, Levallois, France

**Keywords:** cisplatin, temozolomide, paediatric phase I/II, CRM, MGMT

## Abstract

Cisplatin may have additive activity with temozolomide due to ablation of the DNA repair protein *O*^6^-alkylguanine-DNA alkyltransferase (MGMT). This phase I/II study determined recommended combination doses using the Continual Reassessment Method, toxicities and antitumour activity in paediatric patients, and evaluated MGMT in peripheral blood mononuclear cells (PBMCs) in order to correlate with haematological toxicity. In total, 39 patients with refractory or recurrent solid tumours (median age ∼13 years; 14 pretreated with high-dose chemotherapy, craniospinal irradiation, or having bone marrow involvement) were treated with cisplatin, followed the next day by oral temozolomide for 5 days every 4 weeks at dose levels 80 mg m^−2^/150 mg m^−2^ day^−1^, 80/200, and 100/200, respectively. A total of 38 patients receiving 113 cycles (median 2, range 1–7) were evaluable for toxicity. Dose-limiting toxicity was haematological in all but one case. Treatment-related toxicities were thrombocytopenia, neutropenia, nausea-vomiting, asthenia. Hearing loss was experienced in five patients with prior irradiation to the brain stem or posterior fossa. Partial responses were observed in two malignant glioma, one brain stem glioma, and two neuroblastoma. Median MGMT activity in PBMCs decreased after 5 days of temozolomide treatment: low MGMT activity correlated with increased severity of thrombocytopenia. Cisplatin–temozolomide combinations are well tolerated without additional toxicity to single-agent treatments; the recommended phase II dosage is 80 mg m^−2^ cisplatin and 150 mg m^−2^ × 5 temozolomide in heavily treated, and 200 mg m^−2^ × 5 temozolomide in less-heavily pretreated children.

Despite increasing cure rates for many paediatric solid tumours, metastatic disease and certain histologies carry a poor prognosis and require new therapies. The use of new cytotoxic agents in children is based on preclinical data and evaluations in adults (Smith *et al*, 1998).

Temozolomide (Temodal®, Temodar®) is an orally administered imidazotetrazinone second-generation alkylating agent that is spontaneously converted to 5-(3-metyltriazen-1-yl)imidazole-4-carboxamide (MTIC) at physiological pH, and therefore does not require hepatic metabolism for activation. Its ability to cross the blood–brain barrier is of special interest with respect to its activity in central nervous system tumours (Patel *et al*, 2003). Temozolomide has shown activity in preclinical models and in clinical trials in patients with malignant gliomas and melanoma (Newlands *et al*, 1992; O'Reilly *et al*, 1993; Bleehen *et al*, 1995). Phase I and II clinical trials in children and adolescents demonstrated tumour responses in malignant gliomas (Estlin *et al*, 1998; Nicholson *et al*, 1998; Lashford *et al*, 2002).

The cytotoxicity and antitumour activity of temozolomide is determined largely by methylation of DNA at the *O*^6^-position of guanine and the mismatch repair system, and is inversely related to the activity of the DNA repair protein *O*^6^-Alkyguanine-DNA alkyltransferase (also known as *O*^6^-methylguanine-DNA methyltransferase (MGMT)) (Karran and Hampson, 1996; Pegg, 2000; Gerson, 2002; Tentori and Graziani, 2002). Modulation of MGMT activity using pseudosubstrate inhibitors such as *O*^6^-benzylguanine (O^6^BG) (Dolan *et al*, 1994) and more recently *O*^6^-(4-bromothenyl)guanine (PaTrin-2) (Middleton *et al*, 2000a) results in depletion of MGMT and increased sensitivity to DNA alkylating or crosslinking agents, including temozolomide, carmustine, lomustine (Dolan *et al*, 1990; Plowman *et al*, 1994; Liu *et al*, 1996; Wedge *et al*, 1996) and cisplatin (D'Atri *et al*, 1995; Fishel *et al*, 2003). The latter is supported by the observation that MGMT expressing cells are more resistant to the toxic effects of cisplatin (Preuss *et al*, 1996). Cisplatin itself has been reported to inactivate MGMT in HeLa cells (Wang and Setlow, 1989) and to increase the sensitivity of human leukemic blasts to triazene compounds (Piccioni *et al*, 1995). In Jurkat cells, decreased MGMT activity was associated with the attenuation of expression of MGMT mRNA (D'Atri *et al*, 2000). These observations have suggested the possibility of a synergistic antitumour activity of cisplatin with temozolomide and have led to a phase I clinical trial of this combination in adult patients with advanced solid malignancies (Britten *et al*, 1999). The combination was not associated with enhanced toxicity and showed activity in patients with carcinomas and sarcoma.

The present report presents the clinical results of a phase I/II trial of the cisplatin–temozolomide combination in paediatric patients with refractory or recurrent solid tumours. The objectives were to define toxicity profiles and maximum tolerated doses (MTDs), and to recommend a safe dose for phase II testing. In order to perform a single study for two patient cohorts, heavily and nonheavily pretreated patients, we chose a two stage, two group continual reassessment method (CRM) design (O'Quigley *et al*, 1999; O'Quigley and Paoletti, 2003). This model-based adaptive experimental design is used in cancer trials as an alternative to more conventional approaches because it provides a combination of efficiency, safety and the use of information obtained from all treated subjects. To date, there is little experience on the use of the CRM in paediatric oncology trials. We also undertook pharmacodynamic measurements of MGMT activity in peripheral blood mononuclear cell (PBMC), because of its possible role in toxicity and response to both cisplatin and temozolomide, and to further examine the effect of cisplatin on MGMT expression.

## PATIENTS AND METHODS

### Patient eligibility

Eligible patients were required to have a cytologically and/or histologically confirmed diagnosis of a malignant solid tumour (except brain stem tumours with clinical and radiological diagnosis), which was refractory or in relapse after standard treatment, and/or for which no effective treatment was available. Two cohorts were distinguished: less heavily treated (cohort A) and heavily treated (cohort B) patients, the latter defined by prior craniospinal irradiation (CSI) and/or high-dose chemotherapy with stem cell rescue. Early in the study, the definition of cohort B was modified by including patients with disease invasion to the marrow. Following increased haematological toxicity, it was reasonable to assign them to cohort B rather than to the initial cohort A. Patients were to be between 6 months and 21 years of age with ECOG or Lansky performance status of ⩽2 or ⩾50%, respectively, and a life expectancy >6 weeks. Patients were required to have adequate haematopoietic function, that is, neutrophils >1.0 × 10^9^ l^−1^ and platelets >100 × 10^9^ l^−1^, satisfactory hepatic function, that is, bilirubin ⩽1.5 upper normal limit (UNL), transaminases ⩽2.5 × UNL, normal renal function including normal creatinine clearance (>70 ml min^−1^/1.73 m^2^), audiogram ⩽grade 2, and no radiotherapy or chemotherapy within the last 4 weeks before study entry (6 weeks for nitrosoureas). Noninclusion criteria included severe infection, symptomatic or evolutive intracranial hypertension, prior treatment with cisplatin ⩾400 mg m^−2^.

Before study enrollment, complete medical history and clinical examination, concomitant treatments, performance status, haematologic and biochemical profile, clotting rates, creatinine clearance, audiogram, ECG, chest-X-ray, and tumour target assessment were ascertained. The study was conducted in six centers in France in accordance with the Good Clinical Practices and approved by the local Ethics Committee. Patient or parents or the legal representative provided a written informed consent, and patient assent was obtained, when appropriate.

### Treatment

Cisplatin was purchased in vials of 100 mg ml^−1^. The calculated dose was diluted in 180 ml m^−2^ of normal saline. On day 0 of treatment, 480 ml m^−2^ glucose 5% and electrolyte infusion solution was administered prior to the cisplatin solution, which was given over 180 min simultaneously with 180 ml m^−2^ 10% mannitol. This was followed by 180 ml m^−2^ of normal saline and 10% mannitol. Temozolomide (Temodal®) was supplied by Schering-Plough Laboratory (Levallois, France) as white-opaque, hard gelatin capsules in strengths of 5, 20, and 100 mg. The calculated doses were rounded to the nearest 5 mg. For children having difficulties in swallowing, it was allowed to open the capsules and administer the drug in apple juice or stewed fruit. Temozolomide was administered p.o. on days 1–5 to patients who fasted for at least 4 h before and 30 min after each dose. Treatment was repeated every 28 days.

Concomitant antiemetic treatments included ondansetron and prednisone i.v. before cisplatin, and ondansetron p.o. on days 1–5 before temozolomide.

In the event of dose-limiting toxicity (DLT) (defined below), the next cycle was delayed and the doses of cisplatin and temozolomide reduced to the next lowest dose level. If neutrophils or platelets at day 28 were <1.0 × 10^9^ or <100 × 10^9^ l^−1^, respectively, the next cycle was delayed until recovery. In the case of hearing loss or reduction of renal function (grade 3) or at a cumulative cisplatin dose of 600 mg m^−2^, cisplatin treatment was discontinued and treatment pursued with temozolomide alone.

### Experimental study design

The overall objective was to determine for each cohort (A and B) the recommended dose level for phase II testing by using a two stage, two group CRM (O'Quigley *et al*, 1999; O'Quigley and Paoletti, 2003). The MTD was defined as the dose level closest to that producing a DLT in one patient in five on average. The starting dose was 80 mg m^−2^ cisplatin and 150 mg m^−2^ day^−1^ temozolomide for 5 consecutive days (level 1), corresponding to 80% of the recommended dose in single treatments, with planned dose escalation to 80/200 (level 2) and 100/200 (level 3), respectively. Patients of both cohorts were initially treated as a single group for dose escalation. After DLT occurred, the design was switched to escalation/de-escalation according to the two sample CRM model. After each new toxicity evaluation, the best estimates of the MTD in the two groups were reassessed. Information obtained at all levels was used in determining the most appropriate level at which to treat the next patient or group of patients. Early determination rules allowed the study to close when, for either of the cohorts, inclusion would not result in allocation to any level other than the one currently in use with a probability close to 0.9 (O'Quigley and Reiner, 1998).

### Toxicity and response evaluation

Toxicity, graded according to NCI common toxicity criteria (NCI-CTC version 2.0), was assessed by clinical and biological examinations at least weekly during a cycle, before each cycle and at the end of treatment. Dose-limiting toxicity was defined as the occurrence of one of the following events: neutropenia grade 4 with documented infection, persistent (more than 7 days) grade 4 neutropenia or grade 3 or 4 thrombocytopenia, thrombocytopenia necessitating platelet transfusions for a period of more than 7 days, or any nonhaematologic grade ⩾3 toxicity (except vomiting in the absence of adequate treatment, fever without infection, mucocitis, or transient, that is, resolved to grade 1 or 0 at the next treatment cycle in the case of hepatic toxicity). Antitumour efficacy was assessed every two cycles and/or at the end of treatment, according to the Response Evaluation Criteria in Solid Tumour (RECIST) criteria of the National Cancer Institute (Therasse *et al*, 2000). Responses were centrally reviewed by two radiologists (DC and AG).

### *O*^6^-alkylguanine-DNA alkyltransferase activity in PBMCs

Blood samples were collected before cisplatin administration (day 0), on day 1 before the first and on day 5 few hours after the last temozolomide dose. Peripheral blood mononuclear cells were isolated by Ficoll separation and resuspended in EDTA/Tris buffer. *O*^6^-alkylguanine-DNA alkyltransferase activity in cell-free sonicates was determined by measuring the amount of radioactivity transferred from [3H]methylated substrate DNA to MGMT under protein-limiting conditions as previously described (Watson and Margison, 2000). Specific activity was calculated as fmol *μ*g^−1^ DNA in the PBMC extract. DNA content was determined using the Picogreen dsDNA quantification kit (Molecular Probes, Invitrogen, Paisley, UK) according to the manufacturer's instructions. For each set of samples, a pellet of human lymphoblastoid cells (Raji) was included as an assay control and the inter- and intra-assay precision for these samples was 4.6 and 3.5%, respectively, over the whole study. In a separate study, replicate PBMC samples from the same volunteer gave an intrasubject variation of 2.1%. The limits of quantification were defined as 2.5 fmol for MGMT and 0.1 *μ*g ml^−1^ for DNA content. Several of the MGMT values in the post-temozolomide samples were below the lower limit of quantification. In these cases where the sample size was very small, but DNA was still quantifiable, it was reasonable to calculate an MGMT specific activity that would be the maximum possible value, rather than presenting a zero value. While the result would give an underestimate of MGMT inactivation, it was considered more appropriate in the context of our hypothesis to present this rather than an overestimate of inactivation. Statistical differences between treatment and toxicity groups were assessed by the nonparametric Mann–Whitney test.

## RESULTS

### Patients' characteristics

Between May 2001 and June 2002, 39 patients (25 males, 14 females) were included and treated in the study; patients' characteristics are summarised in Table 1. The median age was 12 years 8 months, patients had a median Lansky or ECOG performance scores of 80% and 2, respectively. Predominant tumour types were brain tumours, malignant mesenchymal tumours and neuroblastoma. A total of 38 patients had received prior chemotherapy and/or radiation therapy, one had surgical tumour resection only. Five patients previously received cerebrospinal irradiation and 11 high-dose chemotherapy with autologous stem cell rescue. Six patients had bone marrow metastases at study entry. A total of 38 patients experienced between one and five tumour relapses, one tumour was refractory to prior treatment. Patient No. 007 enrolled in cohort B was deemed nonassessable for toxicity, as he died due to tumour progression within 18 days after treatment start.

### Study treatment and compliance

In total, 39 patients received a total of 114 treatment cycles. In cohort A, 73 cycles were administered in 25 patients at three dose levels, and in cohort B, 42 cycles in 14 patients at two dose levels, in both cohorts, between one to seven cycles per patient (median 2). Cycles were started at days 26–65 (median 29). In cohort A, 16% of cycles (12 out of 73) were delayed by more than 7 days, 12% (nine out of 73) were administered with a 17–40% reduced temozolomide and/or cisplatin dose, in all but one due to haematological toxicity. In cohort B, 17% of cycles (seven out of 41) were delayed, 7% (three out of 41) introduced with a 33% reduced temozolomide dose. The median delay did not increase with treatment duration.

### Dose-limiting toxicity and MTD

In total, 25 patients were evaluable for toxicity in cohort A, and 13 in cohort B. In all but one, DLT during the first cycle was haematological (Table 2). Overall, eight children experienced persistent grade 3 thrombocytopenia, six of them needed platelet transfusion support for a period of more than 7 days. Persistent grade 4 neutropenia was noted in three patients. One child had documented febrile infection during a persistent grade 4 neutropenia; one patient experienced grade 3 septicemia without neutropenia. According to the CRM, the recommended doses for less-heavily pretreated children were defined as cisplatin 80 mg m^−2^ and temozolomide 200 mg m^−2^ day^−1^ × 5, and cisplatin 80 mg m^−2^ and temozolomide 150 mg m^−2^day^−1^ × 5 for heavily pretreated children.

### Dose escalation using the CRM

**Figure 1:**
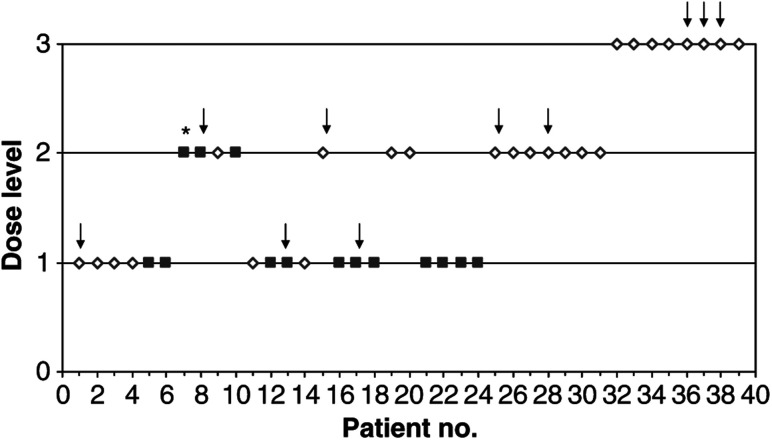
Dose escalation according to the CRM. Patients in cohort A (open diamond) and cohort B (closed square) were treated at the dose levels indicated. Arrows indicate observed DLTs, ^*^ indicates that the patient was not evaluable for toxicity and was excluded from the CRM.

Figure 1 shows dose level and DLT in all treated patients. Patients of both cohorts were initially treated as a single group for dose escalation. The definition of cohort B was refined after patient 13. This modified the group assignment of previously included patients. The third DLT was no longer assigned to cohort A but to cohort B. Running estimates of MTD for both cohorts were updated; dose level 1 was recommended for patient 15 from cohort A and level 2 for patient 16 from cohort B. Patient 28 was first considered as a non-DLT, and dose level 3 was recommended. Had patient 28 been correctly evaluated at that time, then the trial would have come to a halt after patient 30. Ultimately, 25 and 14 patients were included in cohort A and B, respectively.

### Haematologic toxicity

Haematological toxicity was common. Grade 3 or 4 neutropenia was experienced by 72% (18 out of 25) of patients and in 45% (33 out of 73) cycles in cohort A and by 61% (eight out of 13) of patients and in 35% (14 out of 40) cycles in cohort B. Grade 4 neutropenia was persistent for more than 7 days in nine of the 15 cycles and five of 11 patients experiencing grade 4 toxicity. Grade 3 thrombocytopenia occurred in 44% (11 out of 25) of patients and in 25% (18 out of 73) cycles in cohort A and in 54% (seven out of 13) of patients and in 23% (nine out of 40) cycles in cohort B. Persistent grade 3 thrombocytopenia was noted in 12 of the 27 cycles and 10 of 18 patients. The frequency of grade 3 thrombocytopenia was correlated with treatment dose levels. The median nadir neutrophil and thrombocyte counts appeared during the fourth week of the treatment cycle. Neither neutropenia nor thrombocytopenia appeared cumulative.

### Nonhaematologic toxicities

Nonhaematologic toxicities were mostly mild or moderate and appeared independent of dose level and study cohort. Table 3 summarises grade 3 and 4 toxicities. The principle toxicities likely to be related to study treatment were acute nausea and vomiting. Despite premedication with ondansetron and corticoids, grade 1–2 nausea and/or vomiting occurred in 25% cycles and 39% of patients, grade 3–4 in 5 and 11%, respectively. Grade 1–3 asthenia was noted in 29% of patients. Three patients experienced four episodes of grade 3 or 4 septicaemias, one of them during a grade 4 neutropenia. Grade 3 renal toxicity was observed in one patient following intensive vomiting and subsequent dehydration. Toxicity recovered to grade 1 at the end of the cycle.

Grade 2–3 hearing loss, evaluated with audiograms, was noted in five (13%) patients at cumulative cisplatin doses of 100–460 mg m^−2^ (median 240 mg m^−2^). All these patients had prior irradiation to a brain stem glioma, medulloblastoma or ependymoma of the posterior fossa. None had signs of renal toxicity. In total, 12 patients included in the study had prior radiation therapy for a brain stem glioma or a tumour of the posterior fossa. One girl with a brain stem glioma treated with five cycles of cisplatin–temozolomide (400 mg m^−2^ cisplatin) had a normal audiogram at the end of the study; the other six patients had no audiogram evaluation after one or two treatment cycles.

Several patients experienced adverse events, including grade 1–4 pain, headache and neurological alterations, that occurred in general within the context of progressive disease (PD) and were considered secondary to their cancer rather than a consequence of cisplatin–temozolomide treatment.

### Efficacy

Five patients experienced partial tumour response (PR). A patient with an anaplastic ganglioglioma that had progressed previously following temozolomide and radiation therapy experienced a PR after six cycles at 80/150 mg m^−2^ and this continued after the seventh cycle with temozolomide alone. Progression occurred 9 months after treatment start. An anaplastic pleomorphic xanto-astrocytoma of the frontal lobe regressed after two cycles at 80/200 mg m^−2^. Surgical resection of the 2 × 2 mm^2^ tumour residue after seven cycles determined no viable tumour cells. However, shortly thereafter the patient relapsed and died due to tumour progression. A PR was observed in a brain stem glioma after one cycle at 100/200 mg m^−2^. Owing to ototoxicity, treatment continued with temozolomide alone until tumour progression occurred 6 months after the study treatment start. In cohort B, two boys with metastatic neuroblastomas previously treated with high-dose chemotherapy and stem cell rescue and a second line of chemotherapy, experienced nonconfirmed PR this being noted in one of the patients by computer tomographical analysis after four cycles. However, an mIBG scan had shown a slight reduction of fixation sites at cycle 2, but PD at cycle 4. The other patient experienced a PR by mIBG scintigraphy after two cycles; however, he progressed with multiple new lesions at cycle 4.

Four patients had stable disease for at least 6 months treated either with cisplatin–temozolomide or cisplatin–temozolomide followed by temozolomide alone: two patients with a brain stem glioma and one with a soft tissue clear cell sarcoma during 6 months, and one patient with a four times relapsing undifferentiated sarcoma during 11 months.

### Follow-up

Two patients with sarcomas and one with an ependymoma are alive at 30, 28 and 27 months after study entry, respectively. One patient was lost to follow-up after 7.6 months. After the end of the study, six patients had continued treatment with temozolomide alone for 2 to 5 months (median 3.5 months). These patients had experienced PR (two) or stable disease or objective clinical response (four) and were taken off study treatment due to auditory toxicity (three cases) or maximal cumulative cisplatin dose (three cases).

### *O*^6^-alkylguanine-DNA alkyltransferase activity in PBMCs

*O*^6^-alkylguanine-DNA alkyltransferase activity was evaluable in 33 patients at least at one time point (Table 4). Baseline MGMT activity (31 patients) varied from 2.8 to 52.5 Fm *μ*g^−1^ DNA with a median of 12.8 Fm *μ*g^−1^ DNA. *O*^6^-alkylguanine-DNA alkyltransferase in 27 patients after cisplatin administration ranged from 3.0 to 44.7 Fm *μ*g^−1^ DNA with a median of 12.4 Fm *μ*g^−1^ DNA; MGMT in 25 patients on day 5 ranged from <0.4 to 31.3 Fm *μ*g^−1^ DNA with a median of 4.4 Fm *μ*g^−1^ DNA. There was a wide range of change in MGMT levels following treatment in individual patients. After cisplatin, while the mean and median MGMT activities were 116 and 102% of baseline levels, respectively, indicating no overall marked change, individual activities varied from 29 to 309% of baseline levels. Interestingly, in the two patients exhibiting the highest pretreatment levels (42.2 and 52.5 Fm *μ*g^−1^ DNA) there was significant reduction after cisplatin treatment to 12.4 and 27.2 Fm *μ*g^−1^ DNA, respectively. Significant (∼50%) reductions were also seen in four other patients with much lower pretreatment levels. Substantial (>50%) increases in MGMT activity were seen in five patients. After treatment with temozolomide for 5 days, the mean and median MGMT activities were at most 65 and 34% of baseline levels, respectively, indicating an overall substantial inactivation of MGMT (*P*=0.0004; Mann–Whitney test). However, again, individual levels varied considerably, ranging from <5 to 310% of pretreatment levels. Thus, in a total of 25 patients with evaluable samples at baseline and at day 5, 17 patients (68%) had MGMT depletion of more than 50%, two patients (8%) had MGMT depletion of less than 50%, three patients (12%) had an increase of MGMT of more than 50% and three patients (12%) had an increase of less than 50%. The greatest increases were seen in those patients with the lowest pretreatment levels, but the reasons for this are not evident. Depletion was seen at all dose levels tested and was independent of the treatment group, response or any other parameters.

**Figure 2:**
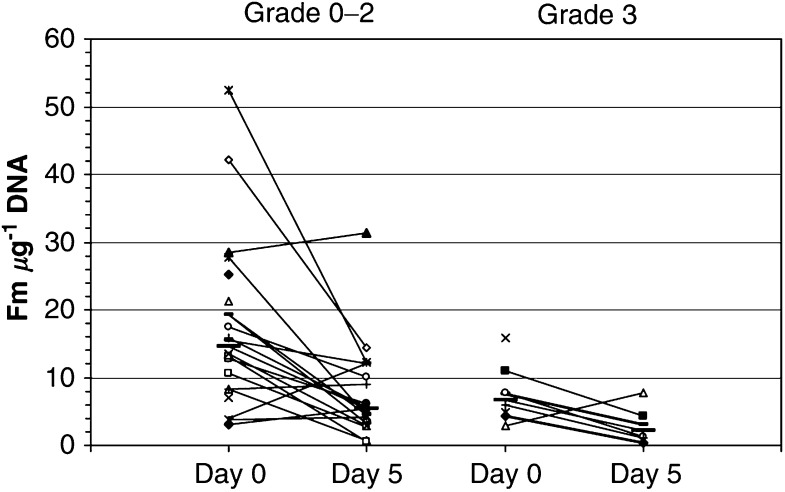
MGMT specific activity (SA: Fm *μ*g^−1^ DNA) in PBMC cells at baseline and depletion at day 5 of cisplatin–temozolomide treatment in samples of patients evaluable for toxicity in relation to grade of thrombocytopenia experienced during the first treatment cycle. The median values for each group are shown as a thick horizontal bar.

In order to assess if there was any correlation between MGMT levels at baseline or after cisplatin–temozolomide treatment and haematological toxicity, we excluded data from Patient No. 007, who was not evaluable for toxicity. Patients experiencing thrombocytopenia grade 0–2 had higher levels of MGMT at baseline (median 14.5 Fm *μ*g^−1^, range 3.1–52.5 Fm *μ*g^−1^) and after CISTEM treatment (median 5.5 Fm *μ*g^−1^, range 0.6–31.3 Fm *μ*g^−1^) than patients with grade 3 toxicity (median 6.7 Fm *μ*g^−1^, range 2.8–15.9 Fm *μ*g^−1^ at baseline; *P*=0.0158; and median 2.2 Fm *μ*g^−1^, range 0.4–7.8 Fm *μ*g^−1^ at day 5; *P*=0.0123; Figure 2). There was no correlation between MGMT and neutropenia: grade 0–2 patients had a median value of 12.8 Fm *μ*g^−1^ (range 3.1–42.2 Fm *μ*g^−1^) and grade 3–4 patients a median of 12.3 Fm *μ*g^−1^ (range 2.8–52.5 Fm *μ*g^−1^) at baseline and a median of 4.6 Fm *μ*g^−1^ (range 0.4–31.3 Fm *μ*g^−1^) and 6.1 Fm *μ*g^−1^ (1.3–12.3 Fm *μ*g^−1^), respectively, on day 5.

In summary, temozolomide treatment for 5 days depleted MGMT in peripheral mononuclear cells in at least 76% of patients and low MGMT activity was correlated with severe thrombocytopenia during the first treatment cycle.

## DISCUSSION

The rationale for combining cisplatin and temozolomide was based on the potential for improved antitumour activity. Both have marked activity in paediatric malignancies; cisplatin is part of many protocols for solid tumours and temozolomide has shown activity in malignant gliomas (Newlands *et al*, 1992; Estlin *et al*, 1998). Both agents target DNA, although they kill cells by different mechanisms: temozolomide via methylation at the *O*^6^ position of guanine and the subsequent action of the mismatch repair system, whereas cisplatin produces DNA crosslinks preferentially via reaction at the N7 positions of guanine and adenine (O'Dwyer *et al*, 1997). Despite this, there is increasing evidence that inactivators of MGMT can enhance the toxicity of cisplatin (Fishel *et al*, 2003). In addition, preclinical studies suggested that cisplatin can attenuate the expression of MGMT (D'Atri *et al*, 2000) resulting in increased sensitivity to temozolomide and additive tumour growth inhibition (Piccioni *et al*, 1995). Although a clinical combination of temozolomide with cisplatin might be beneficial in terms of tumour responses, it is also possible that normal tissue toxicities may be increased, and this needed to be examined in phase I studies in the paediatric setting.

Based on the preclinical findings, in the present paediatric trial, cisplatin was administered as a 3-h infusion prior to temozolomide for 5 days. As the toxicity profiles of cisplatin and temozolomide are very distinct, we started the dose escalation of each agent at 80% of their single agent dose. This selection was appropriate as the results showed that the combination of the two drugs did not potentiate existing toxicities of the individual agents. The DLT of cisplatin–temozolomide after the first treatment cycle in children and adolescents was haematologic. This was consistent with the phase I data of the cisplatin–temozolomide combination in adult malignancies (Britten *et al*, 1999), but also similar to paediatric data for temozolomide given as a single agent (Estlin *et al*, 1998; Nicholson *et al*, 1998; Lashford *et al*, 2002). Subsequently, the MTD of the cisplatin–temozolomide combination for both cohorts (A and B) were similar to those of the previous studies. Britten *et al* (1999) determined the recommended dose for the adult phase II study as temozolomide 200 mg m^−2^day^−1^ × 5 days and cisplatin 75 mg m^−2^ every 28 days. In contrast to our study, cisplatin was scheduled at day 1 beginning 4 h after the first of five daily doses of temozolomide. The paediatric phase I studies of the Children's Cancer Group and the UK group using temozolomide as single agent had stratified according to prior CSI and determined the MTD of temozolomide at 215 mg m^−2^/day × 5 administered every 21 days or 200 mg m^−12^/day × 5 every 28 days for children and adolescents in the non-CSI group and 180 mg m^−2^ day^−1^ × 5 in the CSI-treated group (Estlin *et al*, 1998; Nicholson *et al*, 1998). Both of these paediatric dose-finding studies did not include patients after high-dose chemotherapy or with bone marrow involvement at study entry in the heavily pretreated cohort. These patients often have reduced thrombocytopoiesis, which may have contributed to the lower dose of temozolomide tolerated by our patients in cohort B.

Non-haematological toxicity in children was mostly mild or moderate and did not differ from those known for both single agents. Owing to the potential renal and auditory toxicity of cisplatin, the study limited the maximal cumulative dose to 600 mg m^−2^, including prior treatment. Nevertheless, we observed five patients with hearing loss of more than 50 db at 4000 or 2000 Hz at lower doses. It is worth noting that all five patients had prior irradiation to the brain including a significant radiation dose to the ear region. Our findings confirmed a reduced maximal tolerated cumulative dose of cisplatin in these children. We did not consider that the association of temozolomide had any contribution to this toxicity. In the study of Britten *et al*, three out of 15 patients experienced grade 3 hearing loss and three further patients grade 2 tinnitus without subjective hearing loss (Britten *et al*, 1999). There was no additional information given on the prior treatment of these patients.

Both temozolomide and cisplatin have potential emetic effects. Nausea-vomiting were observed in half of the patients despite premedication; however, they were mostly mild to moderate and manageable with standard antiemetic treatment. The lack of additive emetic effects of the cisplatin–temozolomide combination had been also reported in adult patients (Britten *et al*, 1999). In addition, Britten *et al* had performed pharmacokinetic studies that showed no pharmacokinetic interactions between the two drugs. Thus, cisplatin and temozolomide can be combined at their MTD without additive toxicity profiles; however cumulative toxicity of cisplatin limits the duration of this combination treatment.

A distinguishing feature of this dose finding study was the use of the CRM. The CRM firstly enabled us to perform a single trial with dose-escalations in two stratified groups. The design allows for a more efficient use of the data so that both groups can be evaluated simultaneously, sharing information and thereby improving overall identification of both MTDs. Secondly, the CRM is expected to provide a more accurate estimate of both MTDs since they are identified on the basis of all collected observations. The CRM determined level 2 for the nonheavily pretreated children and level 1 for the heavily pretreated children as recommended doses for the combination. The classical dose escalation would have determined level 1 and minus 1, respectively, which are below the single MTD doses of temozolomide for these cohorts. Thus, the classical design would have been too conservative in its estimate of MTDs. Discussion is ongoing about the accuracy of the dose-finding methodologies used in phase 1 trials (Korn *et al*, 1999; O'Quigley, 1999). The currently ongoing phase II trial in malignant glioma, which is using the doses defined by the CRM, will give further clarification for this combination treatment.

Encouraging tumour responses were observed in children with recurrent gliomas and brain stem gliomas. The response of an anaplastic ganglioglioma in a girl who was one of three siblings of a family with a Li–Fraumeni syndrome with an identified p53 mutation (R273H) was interesting as she had progressed previously with temozolomide and irradiation treatment. P53 mutations and lack of p21-mediated cell arrest have been suggested to render cancer cells resistant to temozolomide (Tentori *et al*, 1998; Hirose *et al*, 2001; Bocangel *et al*, 2002), although the data on the implication of p53 in temozolomide resistance are not consistent (Middlemas *et al*, 2000). Recently, the results of phase II studies combining cisplatin and temozolomide in adult patients with malignant gliomas were published. For 20 patients with recurrent glioblastomas and 13 with anaplastic astrocyctomas, Silvani *et al* (2003) reported progression-free survival at 12 months of 14 and 17%, respectively. Brandes *et al* (2004) reported responses in relapsing chemotherapy-naive glioblastomas in 10 of 49 assessed patients and 34% progression-free survival at 6 months. These findings and those of the present study have resulted in a phase II study in children and adolescents with newly diagnosed and relapsing high-grade gliomas (SFCE and UKCCSG).

A number of clinical trials involving temozolomide and related alkylating agents have included assessments of the levels of expression and depletion of the DNA repair protein MGMT and the majority of studies have examined effects in PBMCs as a surrogate tissue. In the present study, baseline PBMC MGMT activity exhibited a very wide range with values between 2.8 and 52.5 (mean 14.1±1.0) Fm *μ*g^−1^ DNA. This was somewhat higher than previous values (Middleton *et al*, 2000b: range 2.2–25.6, mean 12.6) but similar to other ongoing studies (range 2.3–51.6, mean 15.5: GPM unpublished results). Another study of adult patients has reported a range of ∼0.6 to ∼19 Fm *μ*g^−1^ DNA and a much lower mean of 6.9±4.7 Fm *μ*g^−1^ DNA (Tolcher *et al*, 2003). Some of these differences may be a consequence of the age or disease status of the study groups.

Cisplatin had no overall effect on PBMC MGMT levels, and although we did observe a significant reduction in those patients with high baseline MGMT activity, reductions were also seen in some patients with intermediate activity, while there were increases in others. To our knowledge, no evaluation of MGMT depletion in PBMCs in patients following cisplatin exists to date so that these observations remain to be confirmed. At present, our results using the current doses and schedule do not support the possibility that MGMT depletion might be consistently achieved by cisplatin in a way that could be exploited clinically, although further studies are warranted.

*O*^6^-alkylguanine-DNA alkyltransferase levels in PBMCs were reduced in most patients at the end of temozolomide treatment at all dose levels although it must be noted that part of this depletion might be due to a delayed effect of cisplatin. Previous studies reporting MGMT in adult PBMCs have also shown a range of extents of depletion by temozolomide. A variety of dose regimen and schedules have been examined but complete depletion of MGMT has been observed in very few patients and very wide variations in depletion are always observed (Lee *et al*, 1994; Gander *et al*, 1999; Middleton *et al*, 2000b; Tolcher *et al*, 2003). The reasons for this have yet to be established.

We did not attempt to correlate the initial levels or depletion of MGMT in PBMCs with tumour response, since this was a phase I combination trial including patients with different malignancies and multiple prior lines of treatment. However, regarding toxicity, we found that the grade of thrombocytopenia during cycle 1 was correlated with low baseline levels of MGMT though this was not evident for neutropenia. Although our evaluations are in a limited number of samples and need to be confirmed and extended by further studies, they are supported by previous studies. Thus, lower MGMT activities in PBMCs have been associated with increased haematological toxicity in a phase II study of temozolomide in adult melanoma patients (Middleton *et al*, 2000b) and inactivation of MGMT was correlated with haematological toxicity in a phase I study of temozolomide (Tolcher *et al*, 2003). Nevertheless, to address the questions of MGMT inactivation by cisplatin in relation to tumour responses and toxicities, it would be necessary to undertake larger studies, ideally in direct comparison with equivalent numbers of patients receiving temozolomide alone.

## CONCLUSION

The combination of cisplatin and temozolomide is well tolerated in children and adolescents, generating no toxicity greater than that of the single agents. The antitumour activity observed in the current study led to the ongoing evaluation in a phase II clinical setting in children with newly diagnosed and recurrent malignant gliomas.

## Figures and Tables

**Table 1:** Characteristics of participants *n*=39

**Stratum**	**A without HDCT^a^**/**CSI^b^**/**BM^c^ (*N*=25)**	**B HDCT/CSI/BM (*N*=14)**	**Overall (*N*=39) (%)**
*Age at study entry*			
Median	12 years 8 months	12 year 1 month	12 year 8 months
Range	1 year 10 months–19 years 11 months	4 years 5 months–18 year 9 months	1 year 10 months–19 years 11 months
			
*Gender*			
Male	12	13	25 (64%)
Female	13	1	14 (36%)
			
Lansky performance status	*N*=12	*N*=7	*N*=19
Median (range)	80% (40–100%)	90% (80–100%)	80% (40–100%)
			
ECOG performance status	*N*=13	*N*=7	*N*=20
Median (range)	0 (0–3)	2 (1–3)	2 (0–3)
			
*Diagnoses*			
Central nervous system (CNS) tumous			*N*=18
Malignant glioma	2	0	2
Anaplastic ganglioglioma	1	0	1
Anapl. pleomorphic xanto-astrocytoma	1	0	1
Brain stem glioma	7	0	7
Oligodendroglioma	1	0	1
Ependymoma	3	0	3
PNET	1	0	1
Medulloblastoma	0	2	2
			
Non-CNS tumours			*N*=21
Neuroblastoma	0	4	4
Rhabdomyosarcoma	1	1	2
Ewing tumours	1	2	3
Neurofibrosarcoma	1	1	2
Other sarcoma (indiff, fusiforme)	3	3	6
Desmoplastic small round cell tumour	1	0	1
Nephroblastoma	0	1	1
Retinoblastoma	1	0	1
Melanoma (CNS metastases)	1	0	1
			
Tumour refractory at study entry	1	0	1
*Tumour relapsing*	24	14	38
Median (range)	1 (1–3)	2 (1–5)	2 (1–5)
			
*Prior therapy*			
Surgery only	1	0	1
Chemotherapy+ radiation therapy	16	9	25
Chemotherapy only	5	5	10
Radiation therapy only	3	0	3
Craniospinal irradiation	0	5	5
High-dose chemotherapy	0	11	11
Bone marrow involvement	0	6	6
Prior cisplatin	*N*=4	*N*=4	*N*=8
Median mg m^−2^ (range)	245 (120–372)	300 (140–400)	245 (120–400)
Prior nephrectomy	0	2	2
			
Patients evaluable for toxicity	25	13	38

a High-dose chemotherapy and peripheral stem cell rescue

b Craniospinal irradiation

c Bone marrow involvement.

**Table 2:** Dose-limiting toxicity at cycle one 38 patients

**Dose level**	**Cohort A**		**Cohort B**	
**CDDP/TMZ mg m^−2^ day^−1^**	**DLT/patients**	**DLT**	**DLT/patients**	**DLT**
80/150	1/6	Platelet transfusion >7 days	2/11	Thrombocytopenia G3 >7 days
				Platelet transfusion >7 days
				
80/200	3/11	Febrile neutropenia G4 and platelet transfusion >7 days	1/2	Neutropenia G4 and platelet transfusion >7 days
		Platelet transfusion >7 days		
		Septicaemia G3		
				
100/200	3/8	Infection during neutropenia G4 and thrombocytopenia G3 >7 days	0	
		Platelet transfusion >7 days		
		Neutropenia G4 >7 days		

**Table 3:** Grade 3 and 4 nonhaematological toxicities

	**NCI grade**	**Cycles (*N*=113)**	**Imputability^a^**	**Total (%)**	**Patients (*N*=38)**	**%**
General: asthenia/fatigue	G3	4	2	3.5	4	10.5
						
Gastrointestinal: nausea-vomiting	G3	3	3	2.7	1	2.6
	G4	3	1	2.7	3	7.9
						
*Infection*						
Infection during neutropenia	G3	1	0	0.9	1	2.6
Septicaemia	G3	2	1	1.8	1	2.6
	G4	1	1	0.9	1	2.6
Urinary tract infections	G3	1	0	0.9	1	2.6
						
*Pain*						
Headache	G4	1	0	0.9	1	2.6
Unspecified	G3–4	2	0	1.8	2	5.3
						
*Neurological*						
Neurological alteration	G3–4	3	0	2.7	3	7.9
Reduced vigilance, hallucination	G3	1	0	0.9	1	2.6
Intracranial hypertension	G3	1	0	0.9	1	2.6
						
*Infection*						
Infection in neutrocytopenia	G3	1	1	0.9	1	2.6
Septicaemia	G3	2	1	1.8	1	2.6
	G4	1	1	0.9	1	2.6
Urinary tract infections	G3	1	0	0.9	1	2.6
						
*Renal insufficiency*						
Creatinaemia	G3	1	1	0.9	1	2.6
						
*Ototoxicity (audiogram)*						
Hypacusis	G3	2	2	1.8	2	5.3

a Events considered certain, probably or possibly related to study medication.

**Table 4:** MGMT activity in peripheral blood mononuclear cells

	**ATase SA (Fm *μ*g^−1^)**			**% Activity remaining following**	
**Patient number**	**Day 0**	**Day 1**	**Day 5**	**Cisplatin**	**Temozolomide**
25#	2.8		**7.8**		**276**
2	3.1	3.7	5.5	119	177
24#	3.9	11.9	**4.1**	309	**107**
7	3.9	5.4	0.85	140	22
20	3.9		12.1		310
13#	4.3	5.1	**0.4**	120	**9**
36	4.9	3.6		74	
39	5.9	6.8	1.1	115	19
9	7.1	9.0		127	
10#	7.5	3.1	**3.1**	41	**41**
38#	7.8	3	**1.3**	38	**17**
6#	8.2	9.0	**0.7**	110	**9**
15	8.3	14.3	9.0	173	109
4	10.7	10.9	2.6	102	25
17#	11.0	5.4	**4.3**	49	**39**
12	12.8	5.5	6.2	43	48
21	12.9				
31#	13.1		**0.6**		**5**
30#	13.5	35	**2.9**	259	**22**
22	15.5	33.3	12	215	77
28	15.9	22.4		141	
14	15.9	14.7	5.5	92	35
3	17.5	15.4	10.1	88	57
18	19.2	44.7	4.6	233	24
16#	19.3	13.4	**3.3**	69	**17**
23	21.2	18.4		87	
29	25.3	33.3		132	
11#	27.7	25.2	**4.4**	91	**16**
33	28.4	24	31.3	85	110
19	42.2	12.4	14.5	29	34
34	52.5	27.2	12.3	52	24
					
Mean	14.4	15.4	6.4	116	65
Median	12.8	12.4	4.4	102	34
Range	2.8–52.5	3.0–33.3	<0.4–31.3	29–309%	5–310%

Values in bold are maximum values for sample in # marked patient.

## References

[bib1] Bleehen NM, Newlands ES, Lee SM, Thatcher N, Selby P, Calvert AH, Rustin GJ, Brampton M, Stevens MF (1995) Cancer Research Campaign phase II trial of temozolomide in metastatic melanoma. J Clin Oncol 13: 910–913 7707118 10.1200/JCO.1995.13.4.910

[bib2] Bocangel DB, Finkelstein S, Schold SC, Bhakat KK, Mitra S, Kokkinakis DM (2002) Multifaceted resistance of gliomas to temozolomide. Clin Cancer Res 8: 2725–2734 12171906

[bib3] Brandes AA, Basso U, Reni M, Vastola F, Tosoni A, Cavallo G, Scopece L, Ferreri AJ, Panucci MG, Monfardini S, Ermani M (2004) First-line chemotherapy with cisplatin plus fractionated temozolomide in recurrent glioblastoma multiforme: a phase ii study of the Gruppo Italiano Cooperativo di Neuro-Oncologia. J Clin Oncol 22: 1598–1604 15117981 10.1200/JCO.2004.11.019

[bib4] Britten CD, Rowinsky EK, Baker SD, Agarwala SS, Eckardt JR, Barrington R, Diab SG, Hammond LA, Johnson T, Villalona-Calero M, Fraass U, Statkevich P, Von Hoff DD, Eckhardt SG (1999) A phase I and pharmacokinetic study of temozolomide and cisplatin in patients with advanced solid malignancies. Clin Cancer Res 5: 1629–1637 10430061

[bib5] D'Atri S, Graziani G, Lacal PM, Nistico V, Gilberti S, Faraoni I, Watson AJ, Bonmassar E, Margison GP (2000) Attenuation of *O*(6)-methylguanine-DNA methyltransferase activity and mRNA levels by cisplatin and temozolomide in jurkat cells. J Pharmacol Exp Ther 294: 664–671 10900246

[bib6] D'Atri S, Piccioni D, Castellano A, Tuorto V, Franchi A, Lu K, Christiansen N, Frankel S, Rustum YM, Papa G (1995) Chemosensitivity to triazene compounds and O6-alkylguanine-DNA alkyltransferase levels: studies with blasts of leukaemic patients. Ann Oncol 6: 389–393 7619755 10.1093/oxfordjournals.annonc.a059189

[bib7] Dolan ME, Chae MY, Pegg AE, Mullen JH, Friedman HS, Moschel RC (1994) Metabolism of O6-benzylguanine, an inactivator of O6-alkylguanine-DNA alkyltransferase. Cancer Res 54: 5123–5130 7923129

[bib8] Dolan ME, Stine L, Mitchell RB, Moschel RC, Pegg AE (1990) Modulation of mammalian O6-alkylguanine-DNA alkyltransferase *in vivo* by O6-benzylguanine and its effect on the sensitivity of a human glioma tumor to 1-(2-chloroethyl)-3-(4-methylcyclohexyl)-1-nitrosourea. Cancer Commun 2: 371–377 2242301 10.3727/095535490820873985

[bib9] Estlin EJ, Lashford L, Ablett S, Price L, Gowing R, Gholkar A, Kohler J, Lewis IJ, Morland B, Pinkerton CR, Stevens MC, Mott M, Stevens R, Newell DR, Walker D, Dicks-Mireaux C, McDowell H, Reidenberg P, Statkevich P, Marco A, Batra V, Dugan M, Pearson AD (1998) Phase I study of temozolomide in paediatric patients with advanced cancer. United Kingdom Children's Cancer Study Group. Br J Cancer 78: 652–661 9744506 10.1038/bjc.1998.555 PMC2063055

[bib10] Fishel ML, Delaney SM, Friesen LD, Hansen RJ, Zuhowski EG, Moschel RC, Egorin MJ, Dolan ME (2003) Enhancement of platinum-induced cytotoxicity by O6-benzylguanine. Mol Cancer Ther 2: 633–640 12883036

[bib11] Gander M, Leyvraz S, Decosterd L, Bonfanti M, Marzolini C, Shen F, Lienard D, Perey L, Colella G, Biollaz J, Lejeune F, Yarosh D, Belanich M, D'Incalci M (1999) Sequential administration of temozolomide and fotemustine: depletion of O6-alkyl guanine-DNA transferase in blood lymphocytes and in tumours. Ann Oncol 10: 831–838 10470431 10.1023/a:1008304032421

[bib12] Gerson SL (2002) Clinical relevance of MGMT in the treatment of cancer. J Clin Oncol 20: 2388–2399 11981013 10.1200/JCO.2002.06.110

[bib13] Hirose Y, Berger MS, Pieper RO (2001) p53 effects both the duration of G2/M arrest and the fate of temozolomide-treated human glioblastoma cells. Cancer Res 61: 1957–1963 11280752

[bib14] Karran P, Hampson R (1996) Genomic instability and tolerance to alkylating agents. Cancer Surv 28: 69–85 8977029

[bib15] Korn EL, Midthune D, Chen TT, Rubinstein LV, Christian MC, Simon RM (1999) Commentary. Stat Med 18: 2691–2692 10521859 10.1002/(sici)1097-0258(19991030)18:20<2691::aid-sim194>3.0.co;2-0

[bib16] Lashford LS, Thiesse P, Jouvet A, Jaspan T, Couanet D, Griffiths PD, Doz F, Ironside J, Robson K, Hobson R, Dugan M, Pearson AD, Vassal G, Frappaz D (2002) Temozolomide in malignant gliomas of childhood: a United Kingdom Children's Cancer Study Group and French Society for Pediatric Oncology Intergroup Study. J Clin Oncol 20: 4684–4691 12488414 10.1200/JCO.2002.08.141

[bib17] Lee SM, Thatcher N, Crowther D, Margison GP (1994) Inactivation of O6-alkylguanine-DNA alkyltransferase in human peripheral blood mononuclear cells by temozolomide. Br J Cancer 69: 452–456 8123472 10.1038/bjc.1994.82 PMC1968858

[bib18] Liu L, Markowitz S, Gerson SL (1996) Mismatch repair mutations override alkyltransferase in conferring resistance to temozolomide but not to 1,3-bis(2-chloroethyl)nitrosourea. Cancer Res 56: 5375–5379 8968088

[bib19] Middlemas DS, Stewart CF, Kirstein MN, Poquette C, Friedman HS, Houghton PJ, Brent TP (2000) Biochemical correlates of temozolomide sensitivity in pediatric solid tumor xenograft models. Clin Cancer Res 6: 998–1007 10741727

[bib20] Middleton MR, Kelly J, Thatcher N, Donnelly DJ, McElhinney RS, McMurry TB, McCormick JE, Margison GP (2000a) O(6)-(4-bromothenyl)guanine improves the therapeutic index of temozolomide against A375M melanoma xenografts. Int J Cancer 85: 248–252 10629085 10.1002/(sici)1097-0215(20000115)85:2<248::aid-ijc16>3.0.co;2-v

[bib21] Middleton MR, Lee SM, Arance A, Wood M, Thatcher N, Margison GP (2000b) O6-methylguanine formation, repair protein depletion and clinical outcome with a 4 hr schedule of temozolomide in the treatment of advanced melanoma: results of a phase II study. Int J Cancer 88: 469–473 11054678

[bib22] Newlands ES, Blackledge GR, Slack JA, Rustin GJ, Smith DB, Stuart NS, Quarterman CP, Hoffman R, Stevens MF, Brampton MH (1992) Phase I trial of temozolomide. Br J Cancer 65: 287–291 1739631 10.1038/bjc.1992.57 PMC1977719

[bib23] Nicholson HS, Krailo M, Ames MM, Seibel NL, Reid JM, Liu-Mares W, Vezina LG, Ettinger AG, Reaman GH (1998) Phase I study of temozolomide in children and adolescents with recurrent solid tumors: a report from the Children's Cancer Group. J Clin Oncol 16: 3037–3043 9738573 10.1200/JCO.1998.16.9.3037

[bib24] O'Dwyer PJ, Johnson SW, Hamilton TC (1997) Cisplatin and its analogues. In Cancer: Principles and Practice of Oncology DeVita VTJ, Hellman S, Rosenberg SA (eds). pp 418–432. New York: Lippincott-Raven Publishers

[bib25] O'Quigley J (1999) Another look at two phase I clinical trial designs. Stat Med 18: 2683–2690 10521858 10.1002/(sici)1097-0258(19991030)18:20<2683::aid-sim193>3.0.co;2-z

[bib26] O'Quigley J, Paoletti X (2003) Continual reassessment method for ordered groups. Biometrics 59: 430–440 12926728 10.1111/1541-0420.00050

[bib27] O'Quigley J, Reiner E. (1998) A stopping rule for the continual reassessment method. Biometrika 85: 741–748

[bib28] O'Quigley J, Shen LZ, Gamst A (1999) Two-sample continual reassessment method. J Biopharm Stat 9: 17–44 10091908 10.1081/BIP-100100998

[bib29] O'Reilly SM, Newlands ES, Glaser MG, Brampton M, Rice-Edwards JM, Illingworth RD, Richards PG, Kennard C, Colquhoun IR, Lewis P (1993) Temozolomide: a new oral cytotoxic chemotherapeutic agent with promising activity against primary brain tumours. Eur J Cancer 29A: 940–942 8499146 10.1016/s0959-8049(05)80198-4

[bib30] Patel M, McCully C, Godwin K, Balis FM (2003) Plasma and cerebrospinal fluid pharmacokinetics of intravenous temozolomide in non-human primates. J Neurooncol 61: 203–207 12675312 10.1023/a:1022592913323

[bib31] Pegg AE (2000) Repair of O(6)-alkylguanine by alkyltransferases. Mutat Res 462: 83–100 10767620 10.1016/s1383-5742(00)00017-x

[bib32] Piccioni D, D'Atri S, Papa G, Caravita T, Franchi A, Bonmassar E, Graziani G (1995) Cisplatin increases sensitivity of human leukemic blasts to triazene compounds. J Chemother 7: 224–229 7562019 10.1179/joc.1995.7.3.224

[bib33] Plowman J, Waud WR, Koutsoukos AD, Rubinstein LV, Moore TD, Grever MR (1994) Preclinical antitumor activity of temozolomide in mice: efficacy against human brain tumor xenografts and synergism with 1,3-bis(2-chloroethyl)-1-nitrosourea. Cancer Res 54: 3793–3799 8033099

[bib34] Preuss I, Thust R, Kaina B (1996) Protective effect of O6-methylguanine-DNA methyltransferase (MGMT) on the cytotoxic and recombinogenic activity of different antineoplastic drugs. Int J Cancer 65: 506–512 8621235 10.1002/(SICI)1097-0215(19960208)65:4<506::AID-IJC19>3.0.CO;2-7

[bib35] Silvani A, Eoli M, Salmaggi A, Lamperti E, Maccagnano E, Broggi G, Boiardi A (2003) Phase II trial of cisplatin plus temozolomide, in recurrent and progressive malignant glioma patients. J Neurooncol 66: 203–208 10.1023/b:neon.0000013479.64348.69 15015788

[bib36] Smith M, Bernstein M, Bleyer WA, Borsi JD, Ho P, Lewis IJ, Pearson A, Pein F, Pratt C, Reaman G, Riccardi R, Seibel N, Trueworthy R, Ungerleider R, Vassal G, Vietti T (1998) Conduct of phase I trials in children with cancer. J Clin Oncol 16: 966–978 9508179 10.1200/JCO.1998.16.3.966

[bib37] Tentori L, Graziani G (2002) Pharmacological strategies to increase the antitumor activity of methylating agents. Curr Med Chem 9: 1285–1301 12052167 10.2174/0929867023369916

[bib38] Tentori L, Lacal PM, Benincasa E, Franco D, Faraoni I, Bonmassar E, Graziani G (1998) Role of wild-type p53 on the antineoplastic activity of temozolomide alone or combined with inhibitors of poly(ADP-ribose) polymerase. J Pharmacol Exp Ther 285: 884–893 9580640

[bib39] Therasse P, Arbuck SG, Eisenhauer EA, Wanders J, Kaplan RS, Rubinstein L, Verweij J, van Glabbeke M, van Oosterom AT, Christian MC, Gwyther SG (2000) New guidelines to evaluate the response to treatment in solid tumors. European Organization for Research and Treatment of Cancer, National Cancer Institute of the United States, National Cancer Institute of Canada. J Natl Cancer Inst 92: 205–216 10655437 10.1093/jnci/92.3.205

[bib40] Tolcher AW, Gerson SL, Denis L, Geyer C, Hammond LA, Patnaik A, Goetz AD, Schwartz G, Edwards T, Reyderman L, Statkevich P, Cutler DL, Rowinsky EK (2003) Marked inactivation of O6-alkylguanine-DNA alkyltransferase activity with protracted temozolomide schedules. Br J Cancer 88: 1004–1011 12671695 10.1038/sj.bjc.6600827 PMC2376384

[bib41] Wang LG, Setlow RB (1989) Inactivation of O6-alkylguanine-DNA alkyltransferase in HeLa cells by cisplatin. Carcinogenesis 10: 1681–1684 2766459 10.1093/carcin/10.9.1681

[bib42] Watson AJ, Margison GP (2000) O6-alkylguanine-DNA alkyltransferase assay. Methods Mol Biol 152: 49–61 10957968 10.1385/1-59259-068-3:49

[bib43] Wedge SR, Porteus JK, May BL, Newlands ES (1996) Potentiation of temozolomide and BCNU cytotoxicity by O(6)-benzylguanine: a comparative study *in vitro*. Br J Cancer 73: 482–490 8595163 10.1038/bjc.1996.85 PMC2074446

